# Regulatory Oversight and Safety of Probiotic Use

**DOI:** 10.3201/eid1611.100574

**Published:** 2010-11

**Authors:** Veena Venugopalan, Kimberly A. Shriner, Annie Wong-Beringer

**Affiliations:** Author affiliations: University of Southern California, Los Angeles, California, USA (V. Venugopalan, A. Wong-Beringer);; Huntington Hospital, Pasadena, California, USA (K.A. Shriner, A. Wong-Beringer)

**Keywords:** Probiotics, dietary supplements, Saccharomyces, fungemia, fungi, safety, bacteria, synopsis

## Abstract

Depending on intended use of a probiotic (drug vs. dietary supplement), regulatory requirements differ greatly. For dietary supplements, premarketing demonstration of safety and efficacy and approval by the Food and Drug Administration are not required; only premarket notification is required. *Saccharomyces boulardii* is a probiotic regulated as a dietary supplement intended for use by the general healthy population, not as a drug to prevent, treat, or mitigate disease. However, since recent increases in incidence and severity of *Clostridium difficile* infection, probiotics have been used to treat recurrent and/or refractory disease in hospitalized patients. *Saccharomyces* fungemia secondary to use of the probiotic has been described for patients who are critically ill, are receiving nutrition enterally, or have a central venous catheter. Before use of a probiotic is considered for hospitalized patients, careful assessment of risk versus benefit must be made. To ensure patient safety, probiotics should be properly handled during administration.

## Abstract 2

*Saccharomyces boulardii* probiotics should be used with caution for management of *Clostridium difficile* infections in hospitalized patients.

Probiotics are defined by the Food and Agriculture Organization of the World Health Organization as live microorganisms that, when administered in adequate amounts, confer a health benefit on the host (*1*). The term probiotic can be subcategorized to include probiotic drugs, probiotic foods (e.g., foods, food ingredients, and dietary supplements), direct-fed microbials (probiotics for animal use), and designer probiotics (genetically modified probiotics) (*2*). In the United States, probiotic products are marketed to a generally healthy population as foods or dietary supplements (*3*).

Recent increases in the incidence and severity of *Clostridium difficile* infection (CDI) have led some clinicians to consider use of probiotics as “drugs,” either alone or in combination with traditional antimicrobial agents for the prevention and treatment of CDI. Several recent reviews have summarized results from clinical studies evaluating the efficacy of probiotics in diarrheal illness (*4*–*12*). Our goal is to highlight the current regulatory oversight for probiotics in the United States, identify potential risk situations associated with their administration, and offer suggestions on practical aspects of probiotic administration to ensure patient safety. This review focuses on *Saccharomyces boulardii* (Florastor; Biocodex Pharmaceutical Laboratories, Gentilly, France) as an example of a probiotic product being used as a “drug” to prevent or treat recurrent CDI, particularly in critically ill patients.

## Regulatory Oversight

Depending on the intended use of a probiotic, whether as a drug or a dietary supplement, regulatory requirements differ. According to the Food and Drug Administration (FDA) definition, a drug is an article intended for use in the diagnosis, cure, mitigation, treatment, or prevention of disease (*13*). If a probiotic is intended for use as a drug, then it must undergo the regulatory process as a drug, which is similar to that of any new therapeutic agent. An Investigational New Drug application must be submitted and authorized by FDA before an investigational or biological product can be administered to humans. The probiotic drug must be proven safe and effective for its intended use before marketing (*14*).

If a probiotic is intended for use as a dietary supplement, it is placed under the umbrella of “foods,” and as such is regulated by FDA’s Center for Food Safety and Applied Nutrition (*15*). A dietary supplement is defined by the Dietary Supplement Health and Education Act (DSHEA) of 1994 as a product taken by mouth that contains a “dietary ingredient” intended to supplement the diet. Supplements must contain >1 of the following dietary ingredients: a vitamin; a mineral; an herb or other botanical (excluding tobacco); an amino acid; a dietary substance for use by persons to supplement the diet by increasing the total dietary intake; a concentrate, metabolite, constituent, extract; or combination of any of the above (*16*).

In contrast to drugs, dietary supplements do not need FDA approval before being marketed. However, manufacturers need to notify FDA before marketing a product. According to DSHEA, the manufacturer is responsible for determining that the dietary supplements that it manufactures or distributes are safe and that any representations or claims made about them are substantiated by adequate evidence to show that they are not false or misleading; the manufacturers need not provide FDA with evidence that substantiates the safety or purported benefits of their products, either before or after marketing. If a dietary supplement contains a new dietary ingredient that was not sold before October 15, 1994, then the manufacturer is required to notify FDA and demonstrate to FDA before marketing why the ingredient is reasonably expected to be safe for use in a dietary supplement. On June 22, 2007, FDA announced a final rule establishing Current Good Manufacturing Practice requirements for dietary supplements. To ensure the identity, purity, quality, strength, and composition of dietary supplements, those who manufacture, package, or hold dietary supplements must follow these regulations (*17*). Also, since implementation of the Dietary Supplement and Nonprescription Drug Consumer Protection Act in 2006, manufacturers and distributors of dietary supplements have been required to record and forward to FDA any directly received reports of serious adverse events associated with use of their products. MedWatch Form 3500A (www.fda.gov/downloads/Safety/MedWatch/HowToReport/DownloadForms/ucm082728.pdf) must be completed by the manufacturer or distributor and submitted to FDA. FDA encourages voluntary reporting of adverse events by healthcare professionals, consumers, or patients on MedWatch Form 3500 (www.fda.gov/downloads/Safety/MedWatch/HowToReport/DownloadForms/ucm082725.pdf) (*18*).

## Claims for Dietary Supplements

The law allows that in addition to nutrient content claims, manufacturers of dietary supplements may make structure/function or health claims for their products. For a structure/function claim, FDA requires that manufacturers’ substantiation is accepted by experts in the field and that the claim is truthful and not misleading. The data substantiating structure/function claims need not be publicly available and need not be disclosed. In general, the level of substantiation and the quality of evidence needed to make a structure/function claim are less than that needed to make a health claim. When a structure/function claim is made, the manufacturer must state in a disclaimer that FDA has not evaluated the claim and that the product is not intended to “diagnose, treat, cure, or prevent any disease”; such a claim can legally be made only with regard to a drug (*19*,*20*).

According to FDA, “health claims describe a relationship between a food, food component, or dietary supplement ingredient, and reducing risk of a disease or health-related condition.” In contrast, a structure/function claim describes the process by which the dietary supplement, conventional food, or drug maintains normal functioning of the body and does not need FDA approval before marketing. The data substantiation requirements for the claims described above vary greatly. Before a health claim is authorized, a petition containing the scientific evidence supporting the claim is reviewed by FDA. The systematic review process for a health claim involves defining the relationship between probiotic and disease and identifying relevant studies supporting the claim. Clinical studies are then rated on the basis of quality and strength of evidence. Only data obtained from studies conducted in healthy populations are evaluated because health claims are usually directed at the general population or certain subgroups (e.g. elderly patients). The data supporting a health claim must be published and therefore apply to any product meeting the criteria for the claim (*21*).

## Global Standards for Evaluation of Probiotics

In 2001, in an attempt to standardize the requirements needed to make health claims regarding probiotic agents, the Joint Food and Agriculture Organization of the United Nations/World Health Organization Expert Consultation on Evaluation of Health and Nutritional Properties of Probiotics developed guidelines for evaluating probiotics in food that could lead to the substantiation of health claims (*1*). The proposed guidelines recommend 1) identification of the genus and species of the probiotic strain by using a combination of phenotypic and genotypic tests as clinical evidence suggesting that the health benefits of probiotics may be strain specific, 2) in vitro testing to delineate the mechanism of the probiotic effect, and 3) substantiation of the clinical health benefit of probiotic agents with human trials. Additionally, safety assessment of the probiotic strain should at a minimum determine 1) patterns of antimicrobial drug resistance, 2) metabolic activities, 3) side effects noted in humans during clinical trials and after marketing, 4) toxin production and hemolytic potential if the probiotic strain is known to possess those properties, and 5) lack of infectivity in animal studies.

The Consultation recommends that specific health claims on labeling material on probiotic food items be allowed when sufficient scientific evidence is available and that the product manufacturer take responsibility for ensuring that an independent third party reviews and evaluates the scientific evidence. Since development of these guidelines, only a few manufacturers have conducted small, randomized, controlled studies in humans to prove efficacy and safety of their products. Until more stringent regulations are in place, when assessing therapeutic potential for a probiotic product, clinicians must weigh the available evidence as outlined above. In addition, the manufacturer should take on the responsibility (albeit not required by law) of providing guidance to consumers or clinicians about the type and extent of safety assessments that have been conducted on its products.

## *S. boulardii* as Probiotic

Since the 1950s, *S. boulardii* has been used internationally and extensively as a probiotic (*22*). *S. boulardii* is a live yeast that has been lyophilized and is available in 250-mg capsules for adults. The probiotic may be prescribed as 1–2 capsules to be taken 1–2×/day (*23*). In the United States, *S. boulardii* is marketed as a dietary supplement. The product package displays the following structure/function claims: 1) maintains the balance of the intestinal flora, 2) keeps intestines functioning well, and 3) promotes intestinal health.

## Use of *S. boulardii* in Patients with CDI

Studies of *S. boulardii* in populations other than the healthy general public have demonstrated its efficacy for reducing recurrence of CDI when used in combination with standard therapy. A multicenter, double-blind, placebo-controlled trial investigated the effects of *S. boulardii* (1 g/d) for 4 weeks in combination with vancomycin (high dose 2 g/d or low dose 500 mg/d) or metronidazole (1 g/day) to patients with either initial or recurrent CDI (*24*). Recurrence rates were 16.7% for patients receiving *S. boulardii* with high-dose vancomycin compared with 50% for patients receiving high-dose vancomycin and placebo (p = 0.04). Rates for recurrent CDI did not differ when *S. boulardii* was combined with either low-dose vancomycin or metronidazole. According to the 2010 guidelines for management of CDI in adults, published jointly by the Society of Healthcare Epidemiology of America and the Infectious Diseases Society of America, no compelling evidence exists to support routine use of probiotics for prevention or treatment of CDI (*25*).

## Infectious Complications after Receipt of *S. boulardii*

Safety of dietary supplements is conducted postmarketing. Therefore, much of the safety data on use of *S. boulardii* as a probiotic “drug” are derived from case reports. *Saccharomyces* fungemia is the most severe complication secondary to administration of the probiotic. *S. cerevisiae* and *S. boulardii* have been referred to in the literature interchangeably and have recently been shown by genetic fingerprinting and gene sequencing to be similar on a genetic level and to possibly share metabolic properties (*26*).

The most comprehensive literature review on incidence of invasive *Saccharomyces* infections was conducted by Enache-Angoulvant et al. (*27*). They identified 91 documented cases of invasive *Saccharomyces* infection in the literature (54 cases of *S. cerevisiae* invasive infections vs. 37 cases of *S. boulardii* fungemia). In particular, patients infected with *S. boulardii* were more likely than patients infected with *S. cerevisiae* to have digestive tract disease (58% vs. 6%; p<0.01), to have intravenous catheters (83% vs. 29%; p<0.0001), and to be hospitalized in an intensive care unit (32% vs. 0.05%, p<0.01). The use of biotherapeutic agents containing *S. boulardii* was associated with 40% of all invasive cases. A previously conducted literature review by Muñoz et al. identified 60 cases of fungemia caused by *S. cerevisiae* (*28*). Of note, 48% of patients with fungemia had received a *S. boulardii* probiotic preparation, and another 8% were near patients who had received these agents. The latter finding suggests that *S. boulardii* administration presents an environmental risk for patients who are not receiving the agents.

When Hennequin et al. investigated air and surface contamination related to the opening of a 500-mg packet of freeze-dried *S. boulardii,* they found that the simple act of opening a packet of *S. boulardii* produced substantial air contamination (*29*). Organisms persisted on the arm of the simulated patient 30 minutes after the product was opened and as long as 2 hours on the surrounding table surface. The hands of the technician who had opened the packet were noted to be highly and persistently contaminated despite vigorous handwashing.

Several factors constitute excessive and undue risk for development of *Saccharomyces* fungemia during probiotic administration. These factors are the patient’s immunocompromised state during critical illness, the potential for live yeast spore contamination of healthcare workers’ hands during preparation of the probiotic capsule for administration, and introduction of live yeast from contaminated hands to catheter sites (and patient’s bloodstream).

## Ensuring Patient Safety

Hospitalized patients for whom clinicians may consider use of a probiotic to manage severe and/or recurrent CDI often have many of the above risk factors for development of fungemia, making administration of *S. boulardii* less than desirable and its routine use unsafe. Guideline experts specifically recommend that administration of *S. boulardii* be avoided for persons who are immunocompromised, are critically ill, or have a central venous catheter (*25*). The Florastor package insert even recommends that patients with a central venous catheter consult a healthcare professional before starting therapy and further mentions that “very rare cases of fungemia have been observed in patients with a central venous catheter.”

Institutional guidelines are needed to address the potential safety issues related to *S. boulardii* use. After the decision is made to use probiotics on the basis of careful risk assessment, we suggest that the following measures be taken: 1) healthcare providers should wear gloves during the handling of probiotic agents for administration, then promptly discard the gloves and properly wash their hands with soap and water, 2) drug capsules should not be opened near patients with central venous catheters because aerosolized spores could cross-contaminate sterile sites (i.e., enter blood through catheter site) of patients receiving the probiotic as well as other patients nearby, 3) enteral administration of *S. boulardii* should be avoided because of the risk for environmental contamination and cross-contamination when the seal of the capsule is opened.

Probiotic products contain different genera, different species, or even different strains of the same species. Although the safety concerns noted here for *S. boulardii* may not be extrapolated to other probiotics such as lactobacilli, bifidobacteria, and others, use of any probiotic dietary supplement as a drug in diseased or immunocompromised populations requires specific evaluation of safety in that population.

## Conclusions

The recent increase in incidence and severity of disease caused by hypervirulent strains of *C. difficile* has prompted some clinicians to prescribe probiotics as drugs in combination with standard antimicrobial drug therapy for these patients. However, clinicians need to be aware that, unlike drugs, these probiotic dietary supplements are not required by FDA to undergo rigorous premarketing evaluations for efficacy or safety.

Albeit rare, serious complications (i.e., fungemia) in other than healthy populations receiving probiotics have been reported in the literature. Specifically, most complications related to the administration of *S. boulardii* have occurred in immunocompromised or critically ill patients or in those who had central venous catheters serving as a portal of entry of organisms from healthcare workers’ contaminated hands to patients’ bloodstream during administration. Careful risk assessment for patients and proper handling of the probiotic during administration need to be conducted before using probiotics as drugs in institutional settings. Vigilant reporting of adverse events resulting from probiotic use is necessary to establish the safety profile of these agents when they are used in other than healthy populations.
